# Dr. Fardel’s Memoir on Softening of the Brain

**Published:** 1842-05-28

**Authors:** Durand Fardel


*Memoir on Softening of the Brain.* By M. Durand Fardel.—The object of this paper is to illustrate the inflammatory nature of the disease. Several observations are detailed in which acute *ramollissement* occurred; and in all there existed redness of the brain, as well as softness of its tissue. This redness was in most instances produced by a partial and well-marked injection of vessels; in others there was sanguineous infiltration, as well as vascular injection. The blood was in general evidently derived from the rupture of vessels, and though it existed in a few cases unaccompanied by any vascular injection, analogy would still lead us to refer it to the same cause. There are indeed but three sources from which hæmorrhage into the tissue of an organ could be derived. It must either result from some alteration in the blood, in which case hæmorrhage ought to occur simultaneously into the tissue of several organs; or it may be produced by some alteration in the coats of the vessels—an hypothesis in this case purely gratuitous, and opposed by the fact that ossification of the arteries of the cranium did not exist in any of the eight observations recorded in the paper. Lastly, cerebral congestion and consequent rupture of vessels would account for the occurrence of the redness which exists at the commencement of softening of the brain, and few will deny that softening of an organ consequent on cerebral congestion is the result of inflammatory action.

When death does not take place during the first stage of ramollissement, the disease passes into the chronic state, and changes in the cerebral structure occur in which three different stages may be noticed.

The first stage is characterized by the disappearance of the redness, without any other alteration in the condition of the softened brain. It is to the disease at this period that the description applies which most writers have given of softening of the brain.

In the second period, the ramollissement undergoes very different modifications, according to the part of the brain which it involves. In the cortical substance of the convolutions it assumes the form of yellow membraniform patches; but in the rest of the brain the softened parts become attenuated, the cellular texture of the cerebral substance becomes exposed and infiltrated with a turbid whitish liquid, which appears to consist of the nervous pulp liquefied. To this condition the author applies the name of cellular infiltration.

In the third period, the softened cerebral substance begins to disappear, and real ulcerations form on the surface of the brain, while in its interior the loss of substance gives rise to excavations which must not be confounded with the traces left by the cerebral hæmorrhage.

It is difficult to state anything positively as to the time occupied by these changes. Sometimes they occur with great rapidity, while in other instances their advance is extremely slow—so slow, indeed, that it would almost appear as though the disease might remain stationary for an indefinite length of time in any one of its stages. The advance of the changes of the brain is usually indicated by a corresponding progress in the gravity of the symptoms, while the arrest of the disease is accompanied by a corresponding improvement in the patient's condition.

There is a further distinction necessary to be made between the cases in which the ramollissement remains circumscribed to its original limits and those in which the disease has extended to surrounding parts, so as to present the appearance of a brain, different parts of which are in different stages of ramollissement. These latter cases have afforded M. Fardel the opportunity of ascertaining the various stages of softening of the brain, which form the subject of this memoir.

The cases related by M. Fardel, and the minute anatomical details into which he enters, do not admit of abstract. The memoir, however, is highly interesting, and will well repay a careful perusal.—*Brit. and For. Med. Rev.*, from *Arch. Gén. de Méd.* Jan. and Feb., 1842.

